# Alzheimer’s disease biomarkers in cerebrospinal fluid are stable with the Elecsys immunoassay to most pre-analytical influencing factors except freezing at -80 °C

**DOI:** 10.1186/s42466-023-00257-5

**Published:** 2023-06-29

**Authors:** Franz Felix Konen, Hannah Benedictine Maier, Alexandra Neyazi, Stefan Bleich, Konstantin Neumann, Thomas Skripuletz

**Affiliations:** 1 Department of Neurology, Hannover Medical School, Carl-Neuberg-Straße 1, 30625 Hannover, Germany; 2 Department of Psychiatry, Hannover Medical School, Carl-Neuberg-Straße 1, 30625 Hannover, Germany; 3 Department of Psychiatry and Psychotherapy, Otto-von-Guericke-University Magdeburg, Leipziger Str. 44, 39120 Magdeburg, Germany; 4 Institute of Clinical Chemistry, Hannover Medical School, Carl-Neuberg-Straße 1, 30625 Hannover, Germany

**Keywords:** Alzheimer´s disease, Cerebrospinal fluid, Biomarker, Amyloid ß1–42, Phospho-tau, Total-tau, Blood contamination, Storage duration, Glass vials, Thawing

## Abstract

**Background:**

Alzheimer´s disease is considered a neurodegenerative disease and is diagnosed by exclusion, while the detection of specific cerebrospinal fluid (CSF) biomarkers, namely amyloid-beta (Aβ) peptides Aβ1–42 (Aß42), phospho-tau (181P; P-tau), and total-tau (T-tau), has been shown to improve diagnostic accuracy. Recently, a new generation of sample tubes (Sarstedt false-bottom tubes) for the Elecsys CSF immunoassay for the determination of Alzheimer´s disease biomarkers in CSF was introduced, promising better measurability. However, the pre-analytic influencing factors have not yet been sufficiently investigated.

**Methods:**

In 29 patients without Alzheimer’s disease diagnosis, CSF concentrations of Aß42, P-tau and T-tau were examined in native CSF and after different influencing interventions using the Elecsys immunoassay test method. The following influencing factors were analyzed: contamination with blood (10,000 and 20,000 erythrocytes/µl CSF), 14-day storage at 4 °C, blood contamination of CSF and 14-day storage at 4 °C, 14-day freezing at -80 °C in Sarstedt tubes or glass vials, 3-month intermediate storage at -80 °C in glass vials.

**Results:**

Both storage at -80 °C for 14 days in Sarstedt false-bottom tubes and in glass vials and storage at -80 °C for 3 months in glass vials resulted in significant decreases in Aß42 (13% after 14 days in Sarstedt and 22% in glass vials, 42% after 3 months in glass vials), P-tau (9% after 14 days in Sarstedt and 13% in glass vials, 12% after 3 months in glass vials) and T-tau (12% after 14 days in Sarstedt and 19% in glass vials, 20% after 3 months in glass vials) concentrations in CSF. No significant differences were found for the other pre-analytical influencing factors.

**Conclusions:**

Measurements of the concentrations of Aß42, P-tau, and T-tau in CSF with use of the Elecsys immunoassay are robust to the pre-analytical influencing factors of blood contamination and duration of storage. Freezing at -80 °C results in significant reduction of biomarker concentrations regardless of the storage tube and must be considered in retrospective analysis.

**Supplementary Information:**

The online version contains supplementary material available at 10.1186/s42466-023-00257-5.

## Introduction

Alzheimer´s disease is considered as a neurodegenerative disorder, which is mainly diagnosed by clinical assessment, neuropsychological testing, and brain imaging as well as exclusion of differential diagnoses [1]. The accuracy of clinical diagnosis of Alzheimer´s disease can be improved by detecting specific cerebrospinal fluid (CSF) biomarkers [2]. It has been shown that amyloid beta (Aβ) peptides Aβ1–42 (Aß42) and Aβ1–40, phospho-tau (181P; P-tau), and total-tau (T-tau) in CSF correlate with amyloid status according to positron-emission tomography (PET) and predict future clinical progression of Alzheimer´s disease [2, 3]. Therefore, the use of these CSF biomarkers has been incorporated into diagnostic research guidelines to allow an accurate and timely diagnosis of Alzheimer´s disease to improve patient care and to advance the investigation of potential treatment approaches [2–5].

When determining CSF biomarkers for Alzheimer´s disease, pre-analytical influencing factors must be considered, since sample handling and storage as well as inter-laboratory variances have been reported to influence the measurement results [6–8]. In particular, Aß42 concentrations may be affected, as the peptide is highly aggregating and surface binding [8, 9]. Therefore, polypropylene tubes are recommended for sampling and following Aß42 determination [8, 9]. Recently, a new generation of sample tubes has been introduced for highly reliable methods such as the Elecsys CSF immunoassays (Roche Diagnostics) [10, 11]. Several studies have already investigated these sample tubes (Sarstedt false-bottom tubes) and described that inter-laboratory variability can be reduced by standardization of CSF collection, avoidance of centrifugation, short transfer duration, and immediate measurement after sample collection [12–14]. However, sample storage and blood contamination of CSF have not been shown to significantly influence the CSF biomarkers when using Elecsys CSF immunoassays [14]. In contrast, low blood contamination has been shown to significantly influence total protein as well as CSF IgM concentrations in CSF [15]. Furthermore, plasma concentrations of Alzheimer´s disease biomarker have been shown to remain stable after thawing, usage of different storage tubes, and tubes transfer [16].

Different studies have already investigated different influencing factors for the determination of CSF biomarkers of Alzheimer´s disease with the Elecsys immunoassay. However, some influencing factors, which are frequently observed in routine laboratory work-up, and thus represent the “real world”, have not been investigated so far. Therefore, the aim of the present study was to investigate the influence of pre-analytical interventions in daily clinical routine possibly determining more accurate threshold values.

## Methods

### Patients

A total of 29 patients presenting to Hannover Medical School (MHH) in 2022 and 2023 were included in this prospective study (inclusion criteria: age > 18 years, written informed consent for study participation, complete diagnostic work-up). Some of these patients suffered from severe, long-lasting or therapy-refractory disease courses of underlying psychiatric disorders, thus comprehensive diagnostics with cerebral magnetic resonance imaging and CSF analysis were carried out to exclude possible underlying neurological diseases.

### Lumbar puncture procedure

Lumbar puncture was performed in seated patients with an atraumatic needle (21 G) and introducer (19 G) after extensive dermal disinfection by the same investigator (FFK). Lumbar puncture was performed after breakfast between 10 and 12 a.m., while CSF analyses were performed within 30 minutes after sampling. CSF for the determination of Aß42, P-Tau and T-Tau was collected in false-bottom polypropylene tubes (Sarstedt, # 63.614.625, Nümbrecht, Germany) designed for this test. The first two ml of the CSF were used for different other analyses. Four samples (2.5 ml) were obtained for the investigation of blood contamination and storage duration from the first patient subset. One sample was used for immediate measurement (“native”), one for measurement after storage (14 days), one for blood contamination and immediate measurement and the last one for blood contamination and measurement after storage (14 days). Three additional samples (2.5 ml) were obtained for the study of freezing as a possible influencing factor from a different subgroup of patients. Two samples were used for the comparison of freezing at -80 °C for 14 days in glass vials vs. Sarstedt false-bottom tubes and one sample for the study of freezing at -80 °C for 3 months. The tube was kept upright during entire course of the study.

### Sample analyses

Samples were analyzed without prior centrifugation. Immediately after sample collection, Alzheimer´s disease CSF biomarkers were measured using Elecsys β-Amyloid (1–42) CSF II, Elecsys Phospho-Tau (181P) CSF, and Elecsys Total-Tau CSF immunoassays on the cobas e 801 analyzer (Roche Diagnostics). We also investigated whether pre-analytical interventions, which are frequently observed in laboratory routine diagnostics, have an influence on Alzheimer´s disease biomarker concentrations. The influence of contamination with 10,000 erythrocytes/µL CSF (n = 5) or 20,000 erythrocytes/µL CSF was investigated (n = 12). In addition, CSF samples with and without blood contamination were stored at 4 °C for 14 days and then compared with baseline values (n = 17). Furthermore, baseline concentrations of Alzheimer´s disease biomarkers were compared with concentrations after intermediate storage in glass vials and freezing at -80 °C (for 3 months) and subsequent thawing (n = 17). Samples intermediately stored in glass vials were transferred to Sarstedt false-bottom tubes to enable the peptide and protein measurement.

Finally, the baseline concentrations of Alzheimer´s disease biomarkers were compared with concentrations after 14-day storage at -80 °C in glass vials or Sarstedt false-bottom tubes (n = 12). Samples stored in glass vials were transferred to Sarstedt false-bottom tubes to enable the peptide and protein measurement.

### Statistical analyses

Statistical analysis was performed using GraphPad Prism (La Jolla, CA, USA; version 5.02). The statistical significance level was set at 5%. The Shapiro-Wilk normality test was used to assess the normal distribution of values. Data were expressed as minimum, maximum (min-max), and mean unless otherwise stated. Fisher´s exact test was used for group comparison of binary variables, and either the Mann-Whitney U-Test or paired t-test was used for metric variables. Longitudinal data were analyzed with the Friedman test with Dunn posthoc test for multiple comparisons.

## Results

### Summary of the already investigated pre-analytical influencing factors and the comparison with different assays

Since several authors have already investigated some pre-analytical factors of CSF concentrations of Alzheimer´s disease biomarkers using the Elecsys immunoassay, their findings have been summarized in supplemental Table 1. In addition, the results of the Elecsys immunoassay were compared with other assays for the determination of Alzheimer´s disease biomarkers in CSF, which are listed in supplemental Table 2.

### Patients

Some of the included patients suffered from psychiatric disorders, as shown in Table 1. No patient was diagnosed with Alzheimer’s disease or other types of dementia at the time of inclusion. Aß42 concentrations below the proposed cut-off of 1,100 pg/ml were found in 21% of patients [17]. These patients suffered from major depressive disorder (n = 4), bipolar affective disorder with a current depressive episode (n = 1) and generalized anxiety disorder (n = 1). Some of these patients had neurocognitive deficits, which were interpreted as being part of the underlying psychiatric disorder or an adverse effect of the used medication, since no further clinical or radiological signs for a dementia were observed. Baseline concentrations of Aß42, P-tau, and T-tau are shown in Table 1.

**Table 1:** Demographic data and baseline Alzheimer´s disease cerebrospinal fluid (CSF) biomarker concentrations

Characteristic	Patients (n = 29)
*Demographic data*	
Females:males	15:14
Age [years], median (min-max)	53 (20–83)
Diagnosis	MDD (n = 14), epilepsy (n = 3), ADHS (n = 2), ALS, amnestic syndrome, bipolar affective disorder, CIDP, generalized anxiety disorder, headache (unspecified), M. Parkinson (idiopathic), MS, PTSD, polyneuropathy (non-inflammatory) (each n = 1)
*CSF biomarker baseline concentrations*	
Aß42 [pg/ml], mean (min-max)	1,349 (484-2,500)
P-tau [pg/ml], mean (min-max)	15.7 (8–50)
T-tau [pg/ml], mean (min-max)	193.4 (80–598)
P-tau/Aß42 concentration quotient, mean (min-max)	0.01 (0.006–0.05)
T-tau/Aß42 concentration quotient, mean (min-max)	0.162 (0.0796–0.654)

AD = Alzheimer´s disease, CSF = cerebrospinal fluid, Aß42 = Amyloid beta 1–42, P-tau = Phospho-Tau (181P), T-tau = Total-Tau, MDD = major depressive disorder, ADHS = attention-deficiency-hyperactivity syndrome, ALS = amyotrophic lateral sclerosis, CIDP = chronic inflammatory demyelinating polyneuropathy, MS = multiple sclerosis, PTSD = post-traumatic stress disorder

### Blood contamination of CSF

**Fig. 1:**
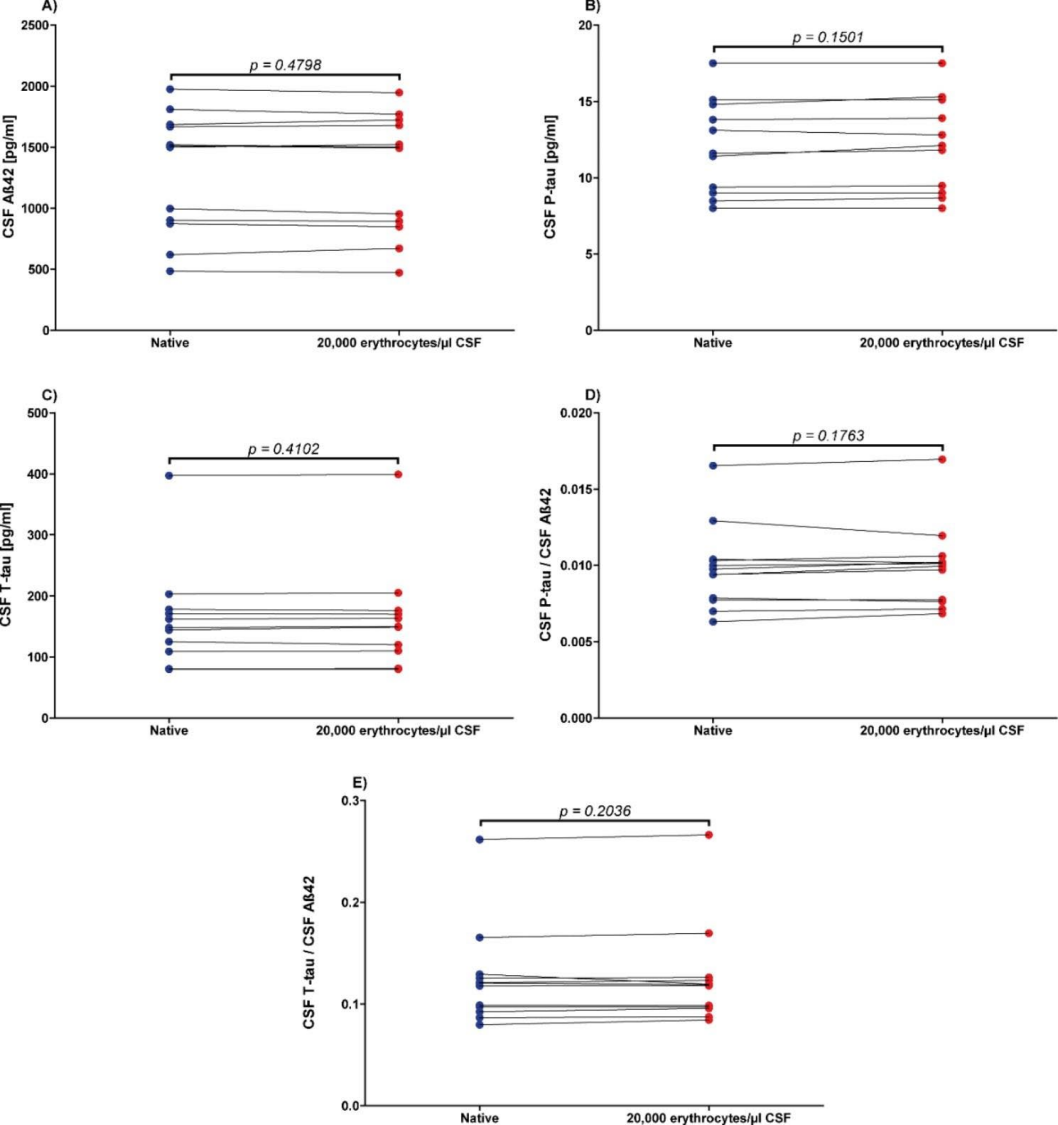
No influence of blood contamination (20,000 erythrocytes/µl CSF) on Alzheimer´s disease cerebrospinal fluid (CSF) biomarker concentrations CSF = cerebrospinal fluid, **A**): Aß42 = Amyloid beta 1–42, **B**): P-tau = Phospho-Tau (181P), **C**): T-tau = Total-Tau, **D**): P-tau/Aß42 concentrations quotient, **E**): T-tau/Aß42 concentrations quotient

Comparison of the baseline concentrations of Aß42, P-tau, and T-tau and the concentration quotients of P-tau/Aß42 and T-tau/Aß42 with the concentrations and quotients after blood contamination with 10,000 erythrocytes/µl CSF (n = 5; p-values between 0.3125 and 0.8125; data not shown) revealed no significant differences. Similarly, blood contamination with 20,000 erythrocytes/µl CSF (n = 12; Fig. 1) did not lead to significant differences in concentrations and quotients compared to baseline (p-values between 0.1501 and 0.4798).

### Storage at 4 °C for 14 days

**Fig. 2:**
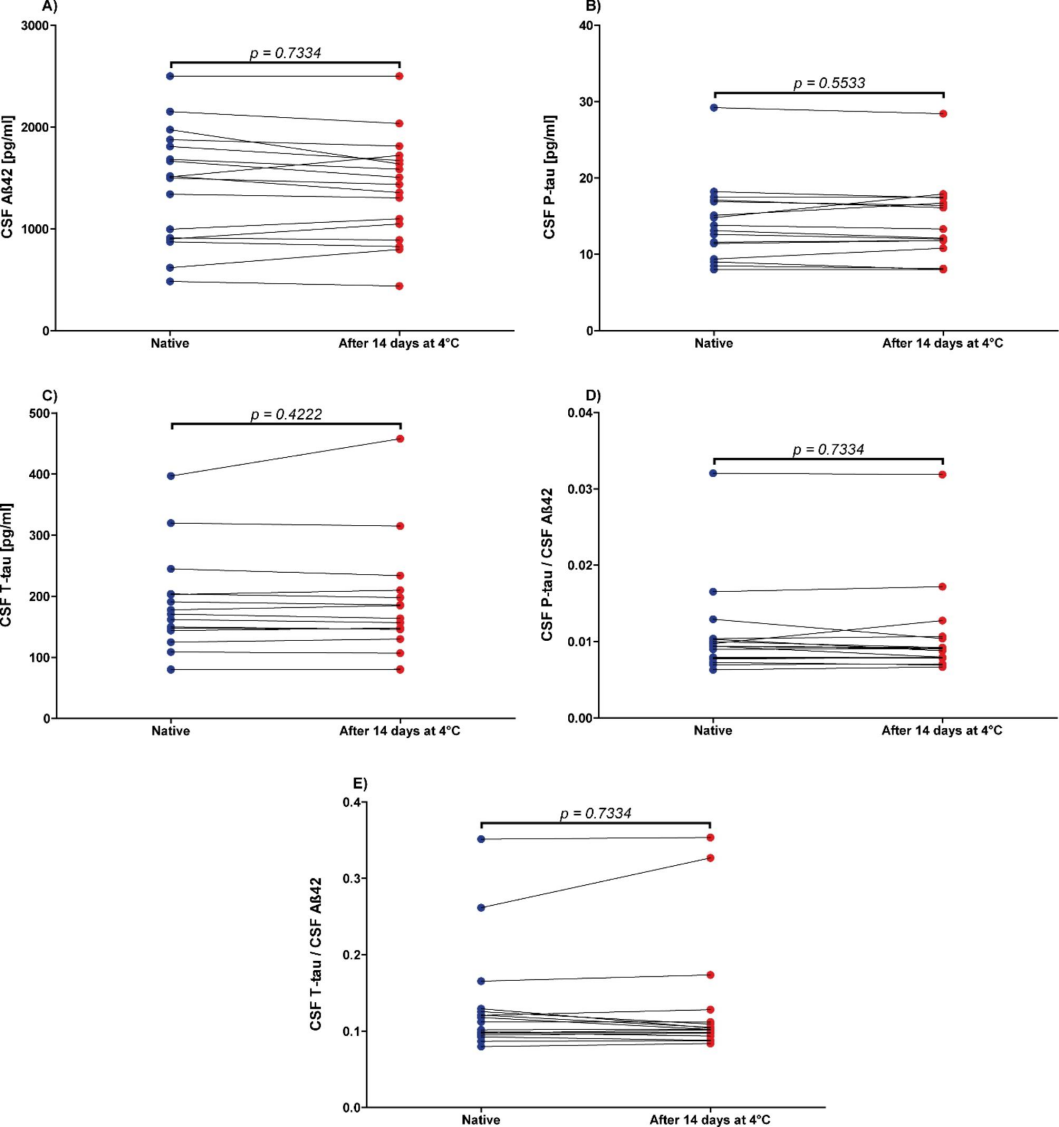
No influence of storage duration (14 days at 4 °C) on Alzheimer´s disease cerebrospinal fluid (CSF) biomarker concentrations CSF = cerebrospinal fluid, **A**): Aß42 = Amyloid beta 1–42, **B**): P-tau = Phospho-Tau (181P), **C**): T-tau = Total-Tau, **D**): P-tau/Aß42 concentrations quotient, **E**): T-tau/Aß42 concentrations quotient

Storage at 4 °C for 14 days did not significantly influence the concentrations of Aß42, P-tau and T-tau, nor the concentration quotients of P-tau/Aß42 and T-tau/Aß42, since no significant differences were observed compared to baseline levels (n = 17; p-values between 0.4222 and 0.7334; Fig. 2).

### Combination of storage at 4 °C for 14 days and blood contamination of CSF

**Fig. 3:**
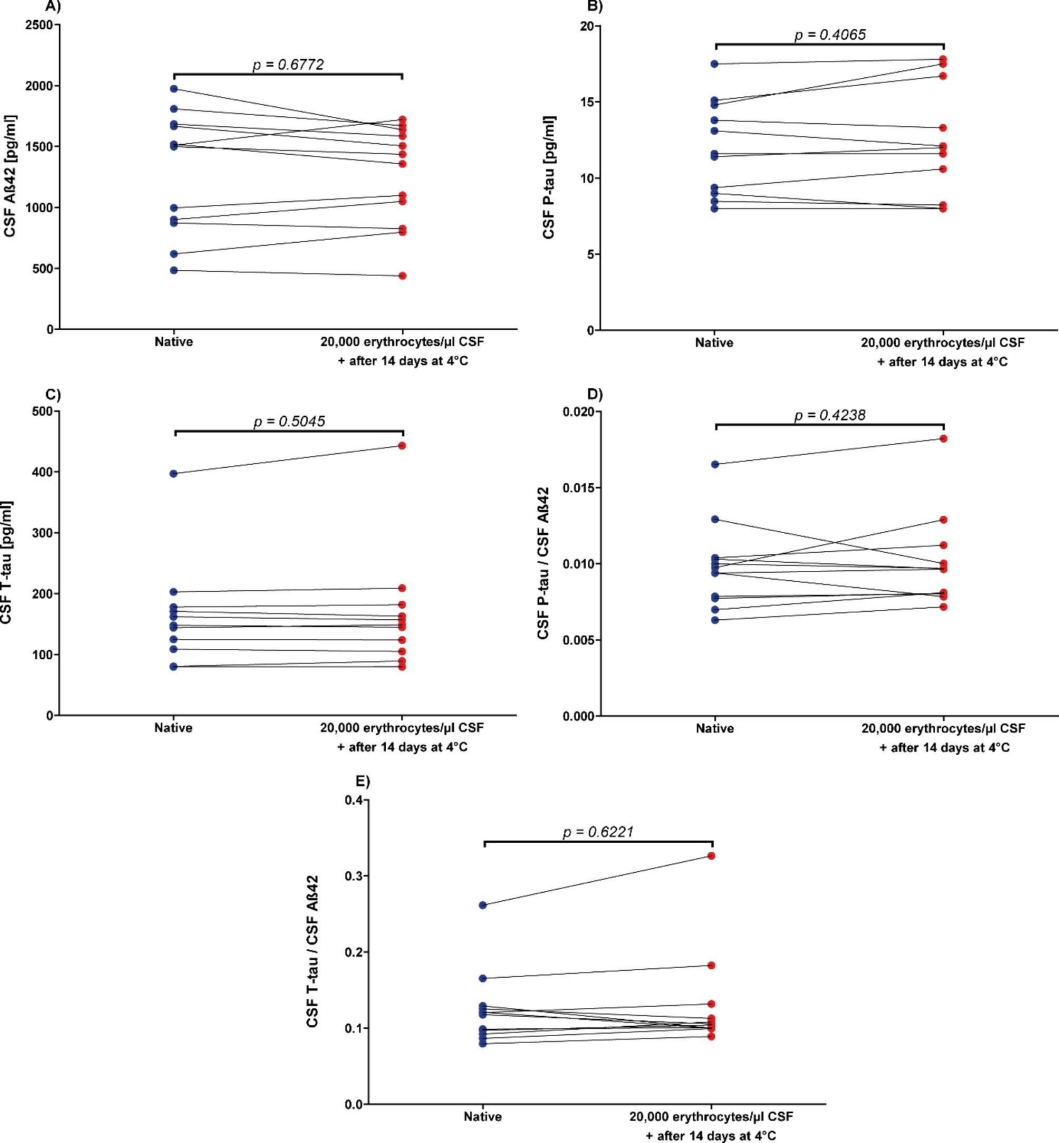
No influence of combining storage duration and blood contamination on Alzheimer’s disease cerebrospinal fluid biomarker (CSF) concentrations CSF = cerebrospinal fluid, **A**): Aß42 = Amyloid beta 1–42, **B**): P-tau = Phospho-Tau (181P), **C**): T-tau = Total-Tau, **D**): P-tau/Aß42 concentrations quotient, **E**): T-tau/Aß42 concentrations quotient

The combination of both influencing factors (storage time of 14 days at 4 °C and contamination with 20,000 erythrocytes/µl CSF) did not result in significantly different concentrations and quotients of Aß42, P-tau, T-tau, P-tau/Aß42 and T-tau/Aß42 (n = 12; p-values between 0.4065 and 0.6772; Fig. 3).

### Storage at -80 °C for 14 days in either Sarstedt false-bottom tubes or glass vials

Storage at -80 °C for 14 days in Sarstedt false-bottom tubes or in glass vials followed by thawing and sample transfer (of samples in glass vials) led to significant concentration differences compared to baseline values.

**Fig. 4:**
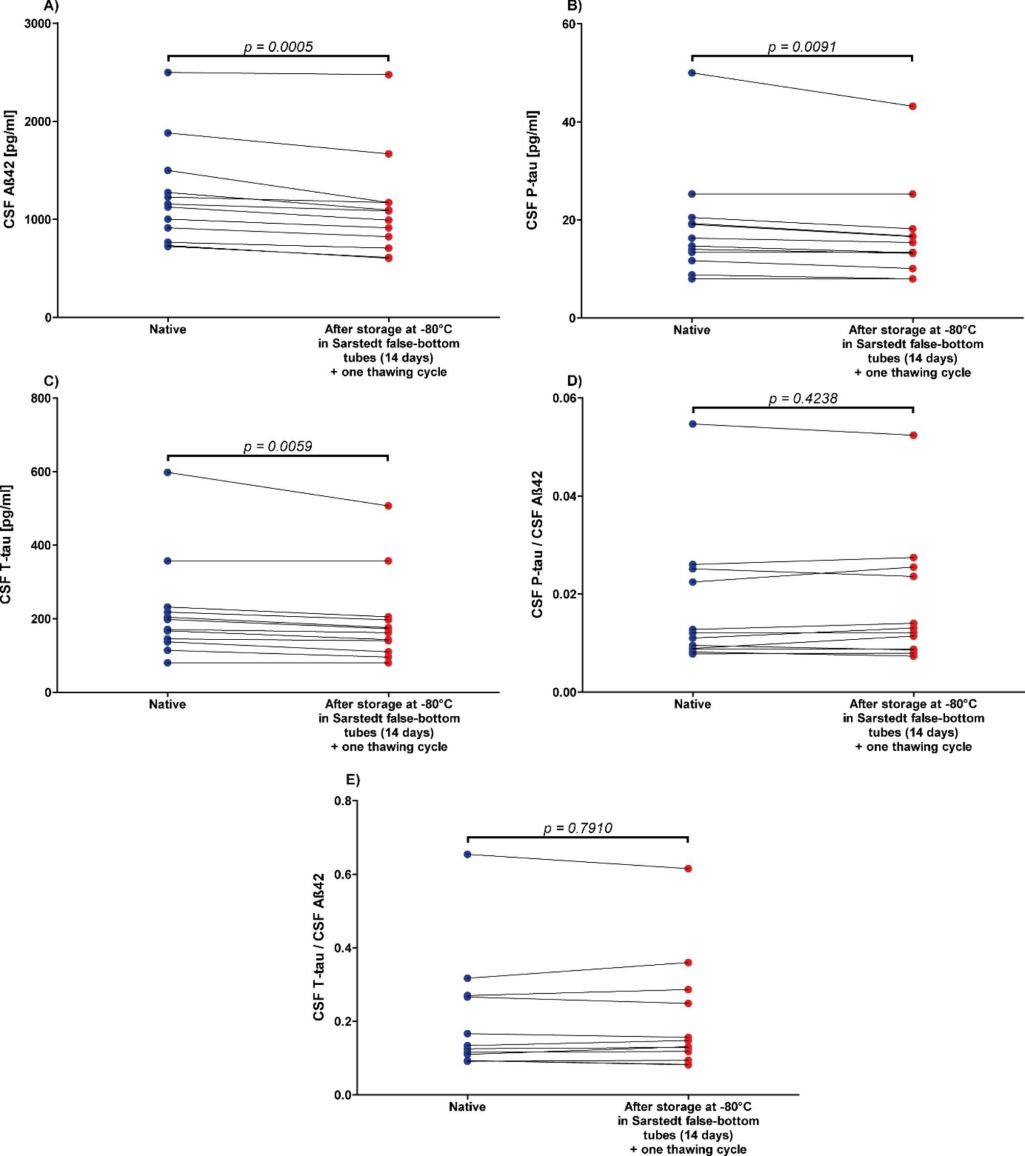
Significant decrease of Alzheimer’s disease cerebrospinal fluid (CSF) biomarker concentrations due to freezing at -80 °C in Sarstedt false-bottom tubes for 14 days CSF = cerebrospinal fluid, **A**): Aß42 = Amyloid beta 1–42, **B**): P-tau = Phospho-Tau (181P), **C**): T-tau = Total-Tau, **D**): P-tau/Aß42 concentrations quotient, **E**): T-tau/Aß42 concentrations quotient

The concentrations of Aß42, P-tau, and T-tau decreased by an average of 13% ±7.5 (p = 0.0005), 9% ±6 (p = 0.0091), and 12% ±7.9 (p = 0.0059), respectively, after storage in Sarstedt false-bottom tubes at -80 °C and thawing (Fig. 4). In contrast, the P-tau/Aß42 and T-tau/Aß42 quotients increased in some cases and decreased in others, thus no significant changes were observed by storage in glass vials compared to baseline (p-values 0.4238 and 0.7910; Fig. 4).

**Fig. 5:**
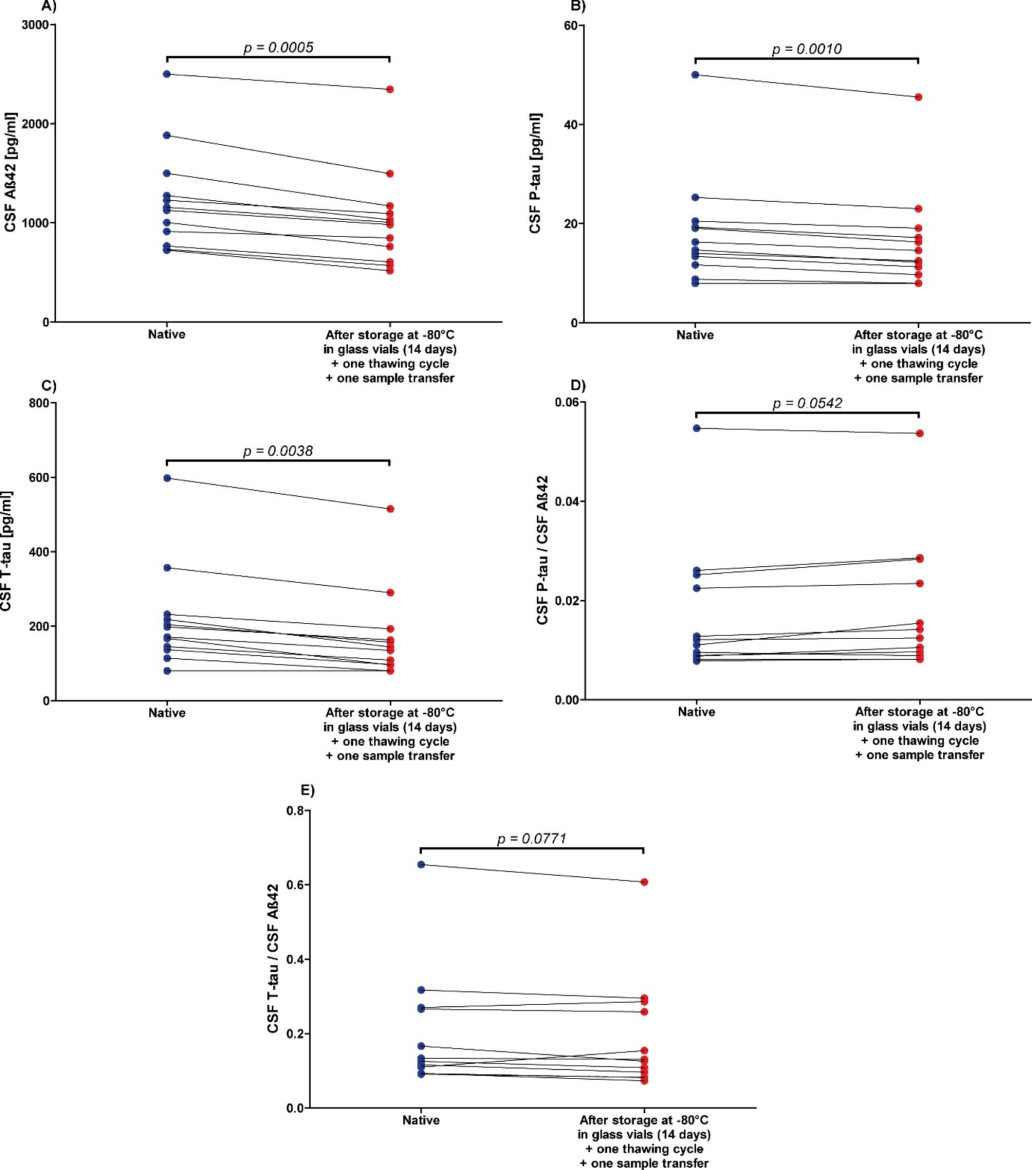
Significant decrease of Alzheimer’s disease cerebrospinal fluid (CSF) biomarker concentrations due to freezing at -80 °C in glass vials for 14 days CSF = cerebrospinal fluid, **A**): Aß42 = Amyloid beta 1–42, **B**): P-tau = Phospho-Tau (181P), **C**): T-tau = Total-Tau, **D**): P-tau/Aß42 concentrations quotient, **E**): T-tau/Aß42 concentrations quotient

The concentrations of Aß42, P-tau, and T-tau decreased by an average of 22% ±10.3 (p = 0.0005), 13% ±6 (p = 0.0010), and 32% ±19.1 (p = 0.0038), respectively, after storage in glass vials at -80 °C, thawing, and transfer into Sarstedt false-bottom tubes for measurement (Fig. 5). In contrast, the P-tau/Aß42 and T-tau/Aß42 quotients partly increased in some cases and decreased in others, so that no significant changes were observed when stored in glass vials compared to baseline (Fig. 5).

A comparison of storage in glass vials or Sarstedt tubes at -80 °C for 14 days showed that the concentrations of Aß42 and T-tau were significantly lower when samples were stored in glass vials (p-values 0.0024 and 0.0020, data not shown). P-tau and the quotients P-tau/Aß42 and T-tau/Aß42 were not significantly different.

### Intermediate storage at -80 °C for 3 months in glass vials

**Fig. 6:**
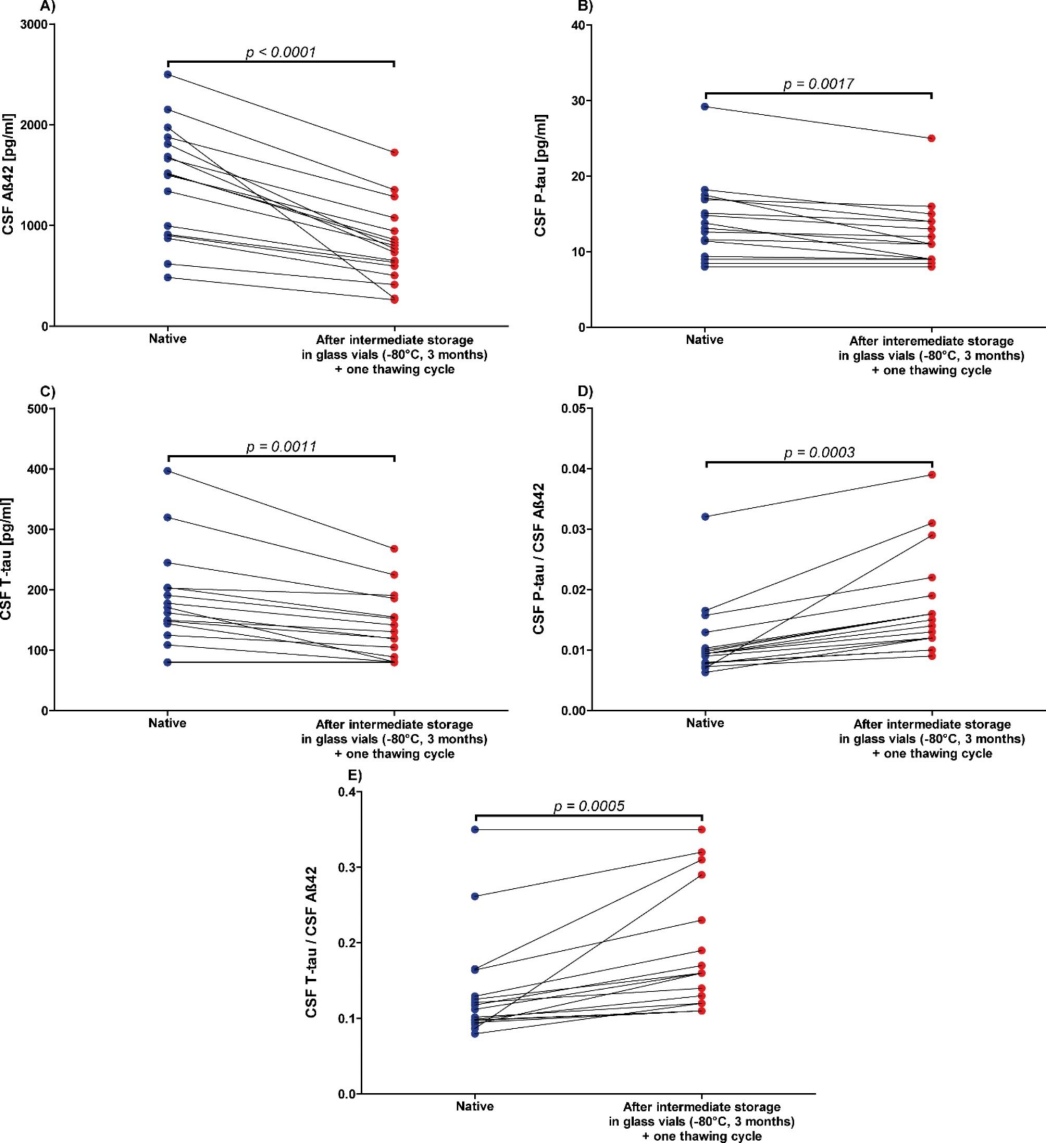
Significant decrease of Alzheimer’s disease cerebrospinal fluid (CSF) biomarker concentrations due to intermediate storage at -80 °C in glass vials for 3 months CSF = cerebrospinal fluid, **A**): Aß42 = Amyloid beta 1–42, **B**): P-tau = Phospho-Tau (181P), **C**): T-tau = Total-Tau, **D**): P-tau/Aß42 concentrations quotient, **E**): T-tau/Aß42 concentrations quotient

Intermediate storage in glass vials and freezing at -80 °C for 3 months, followed by thawing and sample transfer into Sarstedt false-bottom tubes for the determination of Alzheimer´s disease biomarkers resulted in significant concentration differences compared to baseline values. Concentrations of Aß42, P-tau, and T-tau decreased by an average of 42% ±13.4 (p < 0.0001), 12% ±11.5 (p = 0.0017), and 20% ±14.2 (p = 0.0011), respectively, after intermediate storage and thawing (Fig. 6). In contrast, the P-tau/Aß42 and T-tau/Aß42 quotients increased significantly by 34% ±14 (p = 0.003) and 26% ±16.6 (p = 0.0005), respectively, after intermediate storage in glass vials for 3 months and one thawing cycle (Fig. 6).

## Discussion

A new generation of sample tubes (Sarstedt false-bottom tubes) for the Elecsys CSF immunoassay of Alzheimer´s disease biomarkers has been introduced recently. The promise of a better measurability, especially of Aß42, with less influence by pre-analytical factors was made, thus relatively high cut-off values for pathological concentrations have been proposed [10, 11, 17]. CSF biomarkers for Alzheimer´s disease revealed good agreement with amyloid PET in the diagnosis of Alzheimer´s disease and are therefore recommended to support clinical diagnosis in daily routine [3]. Furthermore, Alzheimer´s disease biomarkers measured by the Elecsys CSF immunoassay in patients with cognitive decline were shown to allow the differentiation between different neurodegenerative pathologies such as Alzheimer´s disease and frontotemporal lobar degeneration [18].

Hansson et al. reported stability of Aß42 CSF concentrations up to a blood contamination of 1% of the sample volume and storage duration of 8 days at room temperature and up to 15 days at 2-8 °C [14]. In the present study, contamination with blood up to 20,000 erythrocytes/µl CSF had no influence on the measured concentrations. The combination of blood contamination of the CSF and storage time did neither lead to significant changes in CSF biomarker levels.

The determination of specific thresholds for blood contamination is important for routine diagnostics since blood contamination of 2,500 erythrocytes/µl CSF is known to refine CSF total protein measurements, whereas 5,000 erythrocytes/µl CSF leads to sophistication of CSF IgM determinations [15]. However, CSF biomarkers of Alzheimer’s disease appear to be relatively stable to blood contamination when using the Elecsys automated assay for Aß42, T-tau, and P-tau. Furthermore, knowledge of the robustness of Aß42, T-tau, and P-tau in CSF to pre-analytical influencing factors is important because these studies are lacking for alternative biomarkers such as neurofilament light chain (NFL) [19].

In addition, Alzheimer´s disease biomarker measurement using the Elecsys immunoassay was described as exceptionally robust and accurate, thus Aß42 determination in plasma was proposed as possible rapid and easily available prescreening tool for Alzheimer´s disease [16, 20–22]. Circadian rhythm, time between sampling and centrifugation, and time between centrifugation and measurement were identified as factors influencing plasma concentrations of Alzheimer’s disease biomarkers [16, 22]. In contrast, plasma concentrations of Alzheimer´s disease biomarkers remained stable despite up to three freeze/thaw cycles, up to five tube transfers, different material of tubes or different size [16, 22]. These results could not be reproduced for Alzheimer biomarkers in CSF in the present study. Storage in Sarstedt false-bottom tubes as well as in glass vials for 14 days at -80 °C and a freeze/thaw cycle (for the glass vials: an additional sample transfer) resulted in a significant reduction of Alzheimer’s disease biomarkers in CSF between 9% and 32% compared to baseline. This finding was even more pronounced for intermediate storage in glass vials for 3 months (reduction of Alzheimer’s biomarkers in CSF between 12% and 42%).

A very high susceptibility of Alzheimer´s disease biomarkers in CSF (especially Aß42) to non-specific adsorption to non-polypropylene materials was already described [23–25]. In line with these findings, significant concentration differences between freezing for 14 days in Sarstedt false-bottom tubes and glass vials were found. Glass vials appeared to particularly affect Alzheimer´s disease biomarkers, while plasma levels were largely unaffected by sampling in glass vials [22–25].

## Conclusion

The use of the Elecsys automated assay for CSF Aß42, P-tau, and T-tau shows robust results on the concentrations of Aß42, P-tau, and T-tau, as well as the concentration quotient of P-tau/Aß42 and T-tau/Aß42, which are not affected by the pre-analytical influencing factors blood contamination of the CSF and storage at 4 °C. However, freezing at -80 °C for 14 days has a significant effect on biomarker concentrations and must be considered in retrospective analysis.

## Electronic supplementary material

Below is the link to the electronic supplementary material.

**Supplemental table 1**. Investigated pre-analytical influencing factors and different analytics involving the Elecsys immunoassay and determination of Alzheimer’s disease biomarkers in cerebrospinal fluid (CSF)

**Supplemental table 2**. Investigated comparisons of different analytics involving the Elecsys immunoassay for the determination of Alzheimer’s disease biomarkers in cerebrospinal fluid (CSF)

## Data Availability

The datasets used and/or analysed during the current study are available from the corresponding author on reasonable request.
